# Reporting of Accelerometry in Health Research: A Scoping Review of Current Guidance

**DOI:** 10.1111/sms.70143

**Published:** 2025-10-15

**Authors:** Grace O. Dibben, Carlos Santillan, Soren Brage, Matthew Buman, Edward Duncan, Malcolm H. Granat, Melvyn Hillsdon, Anne Martin, Charles E. Matthews, Paul McCrorie, Rod S. Taylor, Tommi Vasankari, Charlie Foster

**Affiliations:** ^1^ School of Health & Wellbeing University of Glasgow Glasgow UK; ^2^ Independent Researcher Glasgow UK; ^3^ MRC Epidemiology Unit University of Cambridge School of Clinical Medicine Cambridge UK; ^4^ College of Health Solutions Arizona State University Tempe Arizona USA; ^5^ Nursing Midwifery and Allied Health Professions Research Unit University of Stirling Stirling UK; ^6^ School of Health and Society University of Salford Salford UK; ^7^ Department of Public Health and Sport Sciences University of Exeter Exeter UK; ^8^ Division of Cancer Epidemiology and Genetics National Cancer Institute Rockville Maryland USA; ^9^ Robertson Centre for Biostatistics University of Glasgow Glasgow UK; ^10^ UKK Institute for the Health Promotion Research Tampere Finland; ^11^ Bristol Medical School University of Bristol Bristol UK

**Keywords:** 24‐h activity, accelerometer, guidance, methods, physical activity, reporting, sedentary, sleep

## Abstract

The use of accelerometers in health research is ubiquitous, but reporting of methods for translating raw acceleration data into movement behavior estimates remains inconsistent. This scoping review aims to identify and summarize existing reporting guidance for accelerometer‐based assessment of physical activity, sedentary behavior, and sleep in health research. We systematically searched seven bibliographic databases up to May 2024 for literature containing guidance on reporting of accelerometry results in health research. We assessed the methodological rigor of reporting guidance development using the AGREE II tool and EQUATOR Network's best‐practice recommendations. A thematic synthesis categorized reporting guidance across four themes: (1) data collection, (2) data management and initial processing, (3) deriving movement behaviors from acceleration data, and (4) summary metrics. Searches retrieved 7739 records, from which 47 publications were included. Most applied evidence synthesis methods (76%), whilst others used consensus workshops or empirical research to generate reporting recommendations. Only 17% described stakeholder involvement, with limited descriptions of their role. We identified 380 items of reporting guidance, which were synthesized into 124 unique items. Reporting guidance was consistent for data collection, data management and initial processing, and variable derivation, but less so regarding which specific metrics to report. Existing reporting guidance for accelerometry in health research is extensive and wide in scope, but varies in methodological rigor and stakeholder involvement. A consolidated and systematically developed framework is needed to enhance the reproducibility and comparability of future accelerometer‐based research, incorporating stakeholder engagement, consensus‐driven methodology, and piloting to maximize uptake.

**PROSPERO Registration:** CRD42021272228

## 1 Introduction

The composition of movement behaviors, including physical activity, sedentary behavior, and sleep, has important implications for health across the lifespan [1]. These behaviors are often conceptualized within a 24‐h framework, recognizing their interdependence; time spent in one behavior comes at the expense of another, and their combined influence on health depends on both the quantity and quality of each behavior [2]. Accelerometry is a common device‐based method for measuring human behaviors [3], offering an affordable and widely accepted tool for use in many research and consumer applications, sometimes in conjunction with other sensing approaches. However, the numerous methodological decisions involved in setting up data collection, initial data processing, and the derivation of behavioral metrics/variables can have critical implications for the resulting estimates of movement behavior and their associations with health outcomes [4]. Therefore, standardized reporting is essential for ensuring the appropriate interpretation of accelerometer data, particularly within scientific journal articles, including reports of randomized trials, observational studies, and their protocols.

Experts in movement behavior assessment have previously developed frameworks and guidance for designing, conducting, and reporting studies involving acceleration measurement [5, 6, 7, 8]. However, few of these previous efforts have focused exclusively on reporting requirements, and guidelines have had limited visibility (such as lack of open access) and uptake (e.g., minimal journal or societal endorsement). One of the most comprehensive prior reviews examined the reporting of accelerometer use in physical activity intervention studies, identifying significant gaps in reporting quality and noting only small improvements over time [5]. The review also provided recommendations for improvement. Since then, more recent studies have applied Montoye et al.'s recommendations to audit current reporting practices, confirming that inconsistencies persist [9, 10]. Moreover, no previous studies have systematically identified or evaluated published reporting guidance itself, leaving an important gap in understanding how reporting frameworks have evolved.

Advances in accelerometer technology, including high‐resolution data capture, along with data processing capability and the development of innovative metrics and machine learning‐derived measures, have expanded both the potential and complexity of accelerometry‐based health research. Transparent reporting of accelerometry is crucial to ensure consistency, reproducibility, and comparability of findings across studies. This allows researchers to draw meaningful conclusions, replicate methods, synthesize results in systematic reviews, and apply evidence effectively in clinical and public health settings.

This scoping review builds upon and expands Montoye et al.'s findings [5]. The objective is to identify, describe, and summarize new reporting guidance published since July 2014, developed to improve the clarity, completeness, and transparency of accelerometry reporting in health research.

## 2 Materials and Methods

This review is reported in line with the Preferred Reporting Items for Systematic Reviews and Meta‐Analyses extension for Scoping Reviews (PRISMA‐ScR) statement (Additional File S1) [11]. The protocol was registered on the International prospective register of systematic reviews (PROSPERO, registration number: CRD42021272228).

### 2.1 Literature Search

A comprehensive search strategy was developed and conducted by an information specialist in August 2021 (example search strategy provided in Additional File S2) and updated in May 2024 to identify published literature that contained guidance and recommendations on reporting accelerometry in health research published since 31 July 2014 within the following bibliographic databases: PubMed, EMBASE, PsycINFO, CINAHL, SPORTDiscus, PEDro, and Scopus. A supplementary search of the EQUATOR Network library of reporting guidance (https://www.equator‐network.org/reporting‐guidelines/) was also undertaken. Reference lists of included studies were manually searched for additional relevant publications. No language restrictions were applied, and all eligible publications were considered regardless of the language of publication.

### 2.2 Study Selection

Search results were exported into EndNote reference management programme and deduplicated. Clearly irrelevant records (e.g., conference abstracts, commentaries, animal or robotics studies, and records unrelated to movement behaviors) were initially removed by a single reviewer (G.O.D.). This was followed by independent title and abstract screening by two reviewers (of G.O.D. and C.S. or J.X.) against the eligibility criteria (detailed in Table 1). Full texts were then obtained and independently screened against the eligibility criteria in duplicate (G.O.D. and C.S.). Discrepancies were agreed upon by consensus or by a third reviewer if necessary.

**TABLE 1:** Inclusion and exclusion criteria.

Criteria	Include	Exclude
Type of guidance	Reporting guidance developed using any methodology (e.g., systematic review, consensus workshop) and presented in a checklist, flow diagram or explicit text	Guidance relating only to accelerometer validity/calibration studies, the design or conduct of trials, or the use of accelerometers with no specific information on reporting
Population	Publications that are related to measuring movement behavior for health research (i.e., physical activity, sleep, sedentary time)	Publications related to studies of animals or machines/robotics
Publication type	Peer reviewed journal articles	Conference abstracts, editorials, commentaries, and reports referring to reporting guidance
Time period	Publications since 31 July 2014 to May 2024	Articles published prior to 31 July 2014 (date of Montoye et al. search) [5]

### 2.3 Data Extraction and Methodological Characteristics

We developed a standardized data extraction form on Microsoft Word. Data were extracted by one reviewer (G.O.D.) and checked for accuracy by a second (C.S.). Extracted data included publication details (title, lead author, year of publication, and country), the methodology used to develop the reporting guidance, and whether the publication was open access. Additionally, all individual items of reporting guidance were extracted and compiled for thematic synthesis.

As this was a scoping review, no formal quality appraisal of included studies was conducted. Instead, because no dedicated tool exists for assessing the quality of reporting guidelines, we used key domains from the Appraisal of Guidelines for Research Evaluation (AGREE) II instrument for assessing the quality of practice guidelines [12], and the EQUATOR network guidance for developing reporting guidelines [13], to guide our data extraction and examine the methodological approaches used to develop reporting guidance. Specifically, we recorded information on:

- Scope and purpose: Whether the study explicitly aimed to develop reporting guidance, or other guidance or recommendations, and whether the guidance is applicable to a particular population, study design, device or outcome.
- Stakeholder involvement and rigor of development: The extent to which relevant stakeholders (e.g., researchers, clinicians, journal editors, device/software developers) contributed to the development of reporting guidance, representation of intended users, application of consensus methods, and whether the guidance was piloted.
- Clarity of presentation: how the reporting recommendations were presented (e.g., text, tables, supplementary files) and whether examples of good practice were provided
- Endorsement: Whether the reporting guidelines were endorsed by any professional bodies, organizations or journals.

### 2.4 Data Synthesis

A thematic synthesis of the identified items of reporting guidance was conducted, where reporting items were systematically coded and categorized by agreement by two reviewers (G.O.D. and C.F.) according to the following predetermined themes: data collection, data management and initial processing, derivation of movement behaviors from acceleration data, and summary metrics. These themes were selected to reflect the sequential stages involved in handling accelerometer data, from collection to generation of final variables.

## 3 Results

**FIGURE 1:**
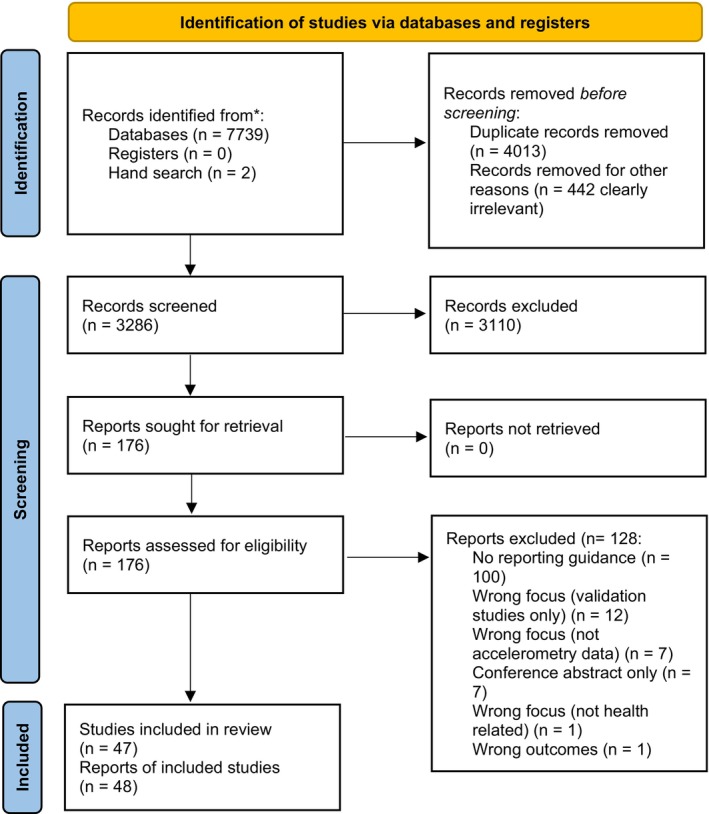
PRISMA flow diagram.

Database and supplementary searches yielded 7741 titles, from which 176 full texts were screened, and 128 were excluded due to a lack of reporting guidance, having the wrong focus, or being conference abstracts only. A total of 48 publications were included in this review (Figure 1) [4, 5, 7, 8, 9, 10, 14, 15, 16, 17, 18, 19, 20, 21, 22, 23, 24, 25, 26, 27, 28, 29, 30, 31, 32, 33, 34, 35, 36, 37, 38, 39, 40, 41, 42, 43, 44, 45, 46, 47, 48, 49, 50, 51, 52, 53, 54, 55]. Two publications reported on the same study [52, 53], resulting in a total of 47 original guidance articles in total.

### 3.1 Characteristics of Included Studies

Table 2 presents an overview of the characteristics of included articles. All were scientific publications, with lead authors based in Europe (24/47, 51%) [8, 10, 15, 18, 19, 20, 21, 24, 25, 26, 27, 28, 32, 39, 41, 42, 43, 44, 45, 47, 49, 50, 51, 54], North America (13/47, 28%) [4, 5, 9, 14, 17, 22, 23, 29, 31, 36, 37, 40, 52, 53], Australasia (9/47, 19%) [7, 16, 30, 33, 34, 35, 38, 46, 55] and Africa (1/47, 2%) [48]. In total, 29 (62%) publications were available open access [7, 10, 16, 18, 19, 20, 21, 24, 25, 26, 27, 28, 29, 30, 31, 33, 37, 38, 39, 40, 41, 45, 46, 47, 49, 50, 51, 52, 53, 54], and the remaining 18 (38%) were restricted access [4, 5, 8, 9, 14, 15, 17, 21, 23, 32, 34, 35, 36, 42, 43, 44, 48, 55].

**TABLE 2:** Characteristics of included studies.

Lead author (year)	Country^a^	Open access	Aim: guidance^b^	Aim: reporting guidance^c^	Methods	Specific study design	Specific population	Specific device	Specific outcome	Guidance format	*N* recommendations
Ancoli‐Israel (2015) [14]	USA	X	✓	✓	NR^d^ manual content developed by sleep/actigraphy experts	X	X	X	Sleep	Text	18
Barry (2015) [15]	UK	X	X	X	Exploratory substudy	X	Older adults	ActiPAL	Ambulation/walking	Text	1
Bei (2015) [16]	Australia	✓	X	X	Systematic review	X	Adults	X	Sleep	Text	1
Benoit (2020) [17]	USA	X	✓	✓	Systematic review	Machine learning	Schizophrenia and bipolar disorder	X	Passive digital phenotyping	Table	28
Blackman (2022) [18]	UK	✓	X	X	Scoping review	X	Adults with mild cognitive impairment	X	Sleep	Table	6
Boerema (2020) [19]	The Netherlands	✓	✓	X	Systematic review	X	Adults	X	SB	Text	3
Breau (2022) [9]	Canada	X	✓	✓	Systematic review	X	Young children	X	PA	Figure	6
Brudy (2021) [20]	Germany	✓	X	X	Systematic review	X	Congenital heart disease	X	PA	Text	7
Casey (2016) [21]	Ireland	X	X	X	Systematic review	X	Multiple sclerosis	X	PA	Text	4
Chan (2022) [22]	USA	✓	✓	✓	Systematic review	X	X	X	PA	Table	3
Clevenger (2022) [23]	USA	X	✓	✓	Case‐study	Hot spot analysis with accelerometer and GPS	Children	X	PA	Table	16
Collins (2019) [24]	UK	✓	X	X	Laboratory study	X	Adults	Garmin Vivosmart 3	Steps and heart rate	Text	1
Crocker (2022) [25]	UK	✓	✓	✓	Systematic review, Delphi surveys^d^	RCTs of PA interventions	X	X	PA	Table	1
Cudejko (2021) [26]	UK	✓	X	X	Systematic scoping review	X	Knee osteoarthritis	X	X	Text	9
Demeyer (2021) [27]	Belgium	✓	✓	✓	Consortium/Task force^d^	X	Chronic obstructive pulmonary disease	X	PA	Text	13
Denneny (2018) [28]	UK	✓	X	X	Rapid review	X	Chronic pain	X	Automated functional data collection	Text	1
Depner (2020) [29]	USA	✓	✓	X	Consensus workshop^d^	X	X	X	Sleep and circadian rhythm	Text and table	2
Diaz (2023) [30]	Australia	✓	X	X	Systematic review	Health interventions	X	X	PA	Text	4
Ellender (2024) [7]	Australia	✓	✓	✓	Literature review^d^ expert subcommittee	X	Adults	X	Sleep	Text	11
Fekedulegn (2020) [31]	USA	✓	X	X	Literature review and methodological demonstration with secondary analysis	X	X	X	Sleep	Text	2
Gabrys (2015) [32]	Germany	X	✓	X	Literature review and consensus procedure (circulating document)^d^	X	X	X	PA	Text	7
Galland (2014) [33]	New Zealand	✓	X	X	Literature review	X	Children	X	Sleep	Text	1
Gilson (2019) [34]	Australia	X	X	X	Systematic review	Observational or interventional	Blue collar workers	X	SB and PA	Table and text	14
Howie (2016) [35]	Australia	X	X	X	Systematic review	RCTs	Children	X	PA	Text	2
Jake‐Schoffman (2019) [36]	USA	X	X	X	Systematic review	RCTs or quasi‐experimental	Adults	X	PA	Text	3
Layne (2015) [37]	USA	✓	X	X	Systematic review	X	Hispanic or Latino populations	X	PA	Tables and text	20
Lee (2021) [38]	Australia	✓	X	X	Scoping review	Interventional	No	Wearable inertial sensor technology with real‐time feedback	Work‐related activities	Table	24
Leeger‐Aschmann (2019) [39]	Switzerland	✓	X	X	Cohort study	X	Children (age 2–6)	ActiGraph	PA	Text	1
Liao (2015) [40]	USA	✓	X	X	Systematic review	X	X	X	PA	Text	1
McArdle (2023) [41]	UK	✓	X	X	Systematic review	X	People with cognitive impairment	X	PA	Figure	3
McGarty (2014) [42]	UK	X	X	X	Systematic review	X	Children and adolescents with intellectual disability	X	PA	Table	17
Migueles (2017) [43]	Spain	X	✓	X	Systematic review	Observational	X	ActiGraph GT3X+	SB, PA, and sleep	Text and figure	9
Migueles (2019) [44]	Spain	X	X	X	Cross‐sectional	X	Children with overweight/obesity	ActiGraph GT3X+	SB and PA	Text	1
Migueles (2021) [45]	Spain	✓	✓	X	Consensus workshop^d^	X	X	X	SB, PA and sleep	Text	2
Montoye (2018) [5]	USA	X	X	X	Systematic review	PA interventions	X	X	PA	Table	13
Mueller (2020) [4]	USA	X	X	X	Secondary analysis of laboratory study	X	Adults	ActiGraph GT3X+	PA	Text	1
Peddle‐Mcintyre (2018) [46]	Australia	✓	✓	✓	Systematic review	X	Cancer patients	X	PA	Text and figure	17
Pontin (2022) [47]	UK	✓	✓	✓	Systematic review	GIS/GPS linkage	X	X	PA	Table	19
Prioreschi (2016) [48]	South Africa	X	X	X	Scoping review	X	Children ≤ 2 years	X	PA	Text	2
Pulsford (2023) [49]	UK	✓	X	X	Systematic review	Observational studies	Adults	X	SB, PA, extrapolation of sleep	Checklist in supplement	24
Schoch (2021) [50]	Switzerland	✓	X	X	Systematic review	X	Children	X	Sleep	Text	6
Thiel (2016) [51]	Germany	✓	X	X	Literature review	X	X	X	PA	Text	14
Tudor‐Locke (2015) [52, 53]	USA	✓	✓	✓	Cross‐sectional	X	Children	X	SB, PA and sleep	Tables in supplement	20
Van Hees (2016) [8]	The Netherlands	X	✓	X	Stakeholder workshop^d^	X	X	X	PA	Table	6
Vetrovsky (2020) [10]	Czech Republic	✓	✓	X	Systematic review	X	Cardio‐vascular disease	X	SB and PA	Table	12
Woelfle (2023) [54]	Switzerland	✓	X	X	Scoping review	X	Multiple sclerosis	X	PA, gait, balance, dexterity or tremour	Text and table	8
Woodforde (2023) [55]	Australia	X	X	X	Systematic review plus exploratory analysis	X	Children and adolescents	X	PA	Text	4

Abbreviations: NR, not reported; PA, physical activity; RCT, randomized controlled trial; SB, sedentary behavior.

^a^ Based on lead author affiliation.

^b^ The study stated generation of guidance as an aim.

^c^ The study stated generation of reporting guidance as an aim.

^d^ Expert or stakeholder involvement was described.

The 47 included articles employed a range of methods to develop their reporting guidance. The majority (35/47, 74%) used evidence synthesis methodology, with five described as literature reviews [7, 31, 32, 33, 51], one rapid review [28], five scoping reviews [18, 26, 38, 48, 54], and 24 systematic reviews [5, 9, 10, 16, 17, 19, 20, 21, 22, 25, 30, 34, 35, 36, 37, 40, 41, 42, 43, 46, 47, 49, 50, 55], one of which included an additional methodological demonstration via secondary analysis [55], and another which incorporated additional Delphi surveys [25]. Four articles described workshop‐based approaches (9%), including two consensus workshops and one consortium/task force initiative [8, 27, 29, 45]. The remaining articles comprised five secondary analyses of accelerometer data collected as part of larger studies and two empirical research studies including a case study and a laboratory study [4, 15, 39, 44, 52, 53]. One did not report its methodology [14].

### 3.2 Methodological Characteristics

#### 3.2.1 Scope and Purpose

Eighteen articles explicitly stated that generating guidance related to accelerometry was an aim of the study. This included guidance on the use of accelerometers [7, 14, 19, 27, 32, 43, 52, 53], a core outcome set for physical activity interventions [25], approaches to data reduction and analysis, and metrics [29, 45], and future directions, challenges, and opportunities [8, 9, 29, 45]. Of these, eleven studies specifically aimed to generate guidance on reporting the application of accelerometry [7, 9, 14, 17, 22, 23, 25, 27, 46, 47, 52, 53].

A number of studies had a specific focus: (1) Population: clinical populations (13/47, 28%) [10, 17, 18, 20, 21, 26, 27, 28, 41, 42, 44, 46, 54], children and adolescents (9/47, 19%) [9, 23, 33, 35, 39, 48, 50, 52, 53, 55], adults (7/47, 15%) [4, 7, 16, 19, 24, 36, 49], older adults (1/47, 2%) [15], blue collar workers (1/47, 2%) [34] or Hispanic and Latino populations (1/47, 2%) [37]; (2) Device: Actigraph (4/47, 9%) [4, 39, 43, 44], ActivPAL (1/47, 2%) [15], Garmin Vivosmart (1/47, 2%) [24], or real‐time inertial sensors (1/47, 2%) [38]; (3) Study design: randomized controlled trials (RCTs) or quasi‐experimental (3/47, 6%) [25, 35, 36], interventional studies (5/47, 11%) [5, 25, 30, 34, 38], observational studies (3/47, 6%) [34, 43, 49], or methodological approaches such as combining accelerometer and GPS data (2/47, 4%) [23, 47], and machine learning (1/47, 2%) [17]; or (4) Outcomes: physical activity (23/47, 49%) [4, 5, 9, 20, 21, 22, 23, 25, 27, 30, 32, 35, 36, 37, 39, 40, 41, 42, 46, 47, 48, 51, 55], sleep (8/47, 17%) [7, 14, 16, 18, 29, 31, 33, 51], sedentary behavior (1/47, 2%) [19], a combination of multiple outcomes (7/47, 15%) [10, 34, 43, 44, 45, 49, 52, 53] or other specific outcomes (6/47, 11%, detailed in Table 2) [15, 17, 24, 28, 38, 54].

#### 3.2.2 Stakeholder Involvement and Rigor of Development

Involvement of stakeholders was described in nine (19%) studies (summary of involvement provided in Additional File S3) [7, 8, 14, 25, 27, 29, 32, 38, 45]. Stakeholders were described as experts and opinion leaders, and included academics, industry partners, physicians, and health professionals, with one study also including lay people [25]. Stakeholder involvement detail was often unclear regarding how many were involved, what level of experience or expertise they had, and explicitly describing what their involvement entailed. Consensus activities were described in five studies (11%) [7, 25, 29, 32, 45]. Delphi methodology was used in one study where professionals and lay people participated in a series of Delphi surveys to rate outcome domains and outcome measures for a core outcome set, ultimately reaching consensus on two domains [25]. Other studies utilized workshops [8, 29, 45] or expert panels [7, 32] to develop recommendations or consensus statements. Again, the level of detail varied, with some studies describing consensus as being reached through discussions or circulation of drafts but did not specify structured methods. None of the studies described a process of pilot testing or refining their reporting guidance based on user feedback or application.

#### 3.2.3 Clarity of Presentation

Guidance was presented in a variety of formats and was not always easily identifiable within the publication (see Table 2). Seventeen studies used tables to present recommendations [5, 8, 10, 17, 18, 22, 23, 25, 29, 34, 35, 38, 42, 47, 49, 52, 53, 54], with four also including recommendations in the main text [29, 34, 37, 54], and two providing tables in supplementary files [49, 52, 53]. Four studies used figures [9, 41, 43, 46], with two providing additional text‐based recommendations [43, 46]. Six studies structured their recommendations under subheadings [7, 14, 27, 31, 32, 51], two studies embedded them within a research agenda list or box [16, 45], and one study used a best‐practice points box [50]. Seventeen studies presented recommendations within their discussion sections [4, 15, 19, 20, 21, 24, 26, 28, 30, 33, 35, 36, 39, 40, 44, 48, 55]. Additionally, examples of good reporting practice, clarifications, definitions, or options were provided in 10 publications [5, 9, 14, 23, 27, 42, 44, 47, 49, 52, 53].

#### 3.2.4 Endorsement

Two guidelines were directly associated with or commissioned by professional societies (Society of Behavioral Sleep Medicine [14] and the Australian Sleep Association [7]). Additionally, three studies described workshops or task forces that were managed, sponsored, or supported by professional societies (COPD Foundation [27], Sleep Research Society [29], and German Association for Sports Science [32]).

### 3.3 Narrative Description of Guidance

Sixteen of the 46 articles (35%) emphasized the need for standardized and transparent reporting in accelerometry studies to improve comparability, reproducibility, and interpretation of findings across studies [10, 20, 21, 22, 28, 29, 32, 33, 34, 36, 37, 40, 42, 45, 46, 54].

**FIGURE 2:**
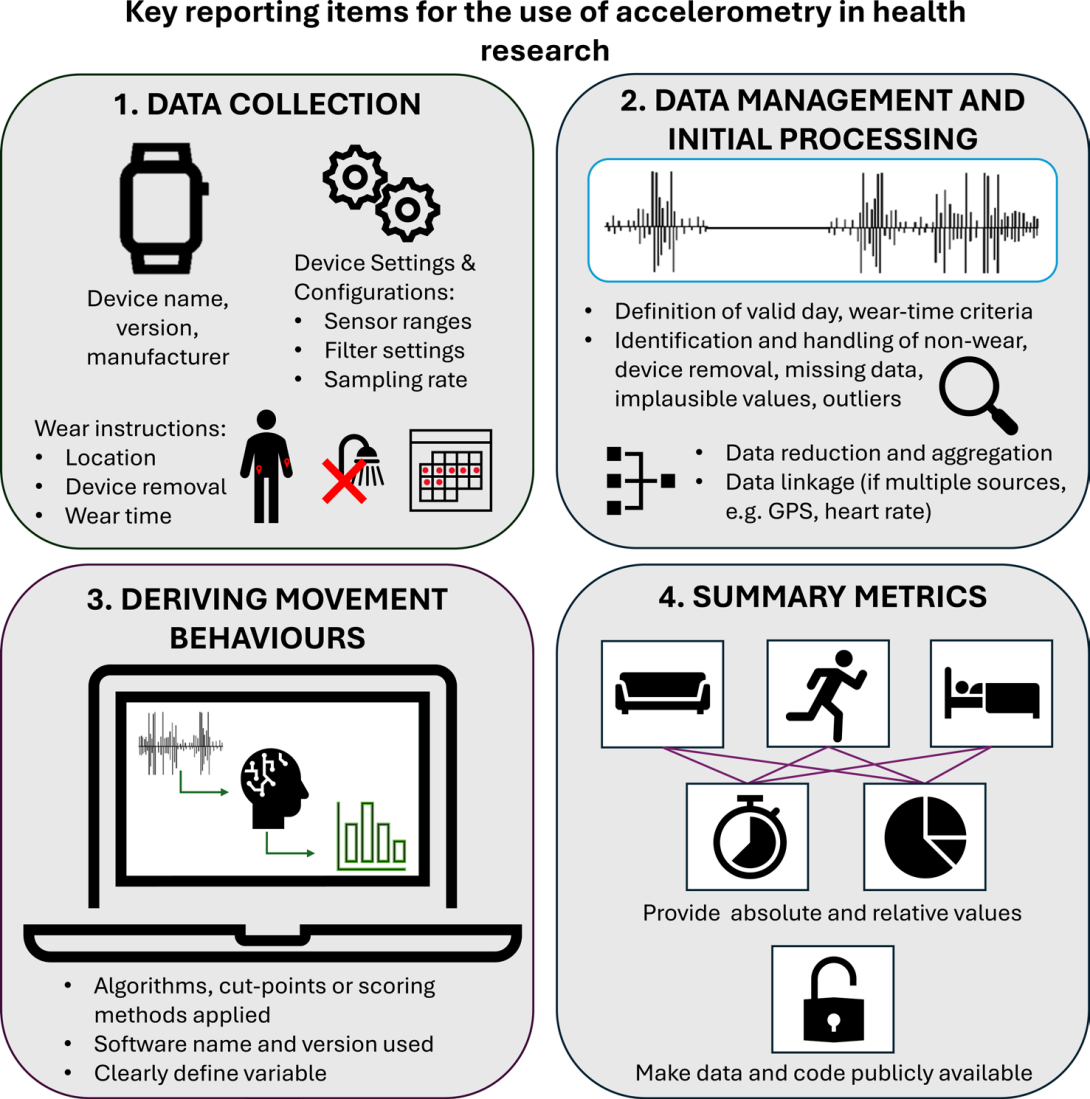
Summary of commonly recommended reporting items for accelerometry in health research, organized by four key themes identified in this scoping review.

The identified reporting guidance covered a wide range of recommendations on how to document and report accelerometry in health research. A total of 380 items of reporting guidance relating to accelerometry were extracted from the included studies (median of 6 items per article, range 1–24). These were synthesized into 124 unique recommended reporting items, which were then categorized into four overarching themes that reflect the key stages of managing accelerometer data for research: (1) data collection, (2) data preparation, (3) deriving movement behaviors from acceleration data, and (4) summary metrics. Figure 2 provides a visual overview of the four themes and commonly recommended reporting items. Full details of reporting recommendations are provided in Additional File S4.

#### 3.3.1 Theme 1: Data Collection

This first theme relates to the decisions and methods involved in collecting accelerometer data from participants. There was strong agreement across 20 articles that key device specifications should be reported, including the device name, model, version, manufacturer, and firmware [5, 9, 10, 14, 17, 19, 23, 24, 26, 27, 29, 37, 38, 46, 47, 49, 50, 51, 52, 53, 54]. Eight articles recommended reporting details of device validity and/or reliability [17, 27, 38, 41, 42, 47, 49, 51]. Additionally, 19 articles emphasized the importance of reporting device settings and configurations, such as sampling rate, epoch length, use of idle mode, and filter settings at device initialisation [5, 9, 10, 14, 20, 21, 22, 23, 26, 27, 32, 37, 38, 43, 46, 47, 49, 50, 51, 54].

For data collection protocols, 14 articles recommended specifying the device placement (e.g., body site, side, and orientation) and attachment method [5, 9, 10, 20, 23, 32, 37, 38, 42, 43, 46, 49, 51, 54]. Sixteen articles recommended detailing the collection period (e.g., number of days, weekdays vs. weekends) [5, 7, 10, 14, 17, 19, 20, 27, 32, 34, 36, 42, 46, 47, 49, 55], with three specifically advising researchers to justify the length of the monitoring period, and if it was < 7 days [34, 42, 55]. Five articles suggested reporting contextual factors including the season of data collection, weather conditions, or participant setting (e.g., home, work, or hospital) [14, 17, 23, 47, 51].

Participant recruitment and device management were also discussed. Six articles recommended documenting device distribution and retrieval methods [5, 10, 38, 42, 46, 49], and five recommended reporting instructions given to participants (e.g., how to wear the accelerometer, removal during bathing) [27, 31, 37, 42, 49], and any strategies to encourage compliance (e.g., reminder calls or financial incentives) [17, 37, 42, 49, 51]. Four articles called for reporting the number of devices distributed and the number of lost or malfunctioning devices [5, 49, 52, 53].

Some specific recommendations related to the use of mobile phones [17], devices that provide feedback to the wearer [38], data privacy laws [17], digital technology literacy [17], and sample size [34].

#### 3.3.2 Theme 2: Data Management and Initial Processing

Theme two relates to the decisions, processing, definitions, and assumptions applied to each accelerometer data record prior to the derivation of the variables of interest. There was strong consensus across 17 articles that wear‐time criteria should be clearly defined, including valid day definitions, and the minimum number of valid days required for inclusion in the final analytical sample [5, 10, 19, 20, 21, 22, 27, 30, 32, 34, 36, 37, 41, 42, 46, 49, 55]. Additionally, 13 articles recommended reporting a summary of the average total wear time or adherence rates across participants [17, 19, 21, 22, 26, 32, 36, 37, 46, 49, 51, 52, 53, 55].

Regarding data completeness, seven articles recommended reporting the procedures for handling missing data (e.g., imputation) [16, 30, 37, 42, 47, 49, 50]. Five articles advised reporting rates of missing data [16, 17, 23, 35, 47], and nine recommended documenting the reasons for data loss or exclusion (e.g., device malfunction, invalid data) [5, 7, 10, 14, 37, 40, 49, 51, 52, 53], with three articles recommending that the impact of missing data on the results and interpretation should be discussed [7, 14, 35]. Eleven articles recommended describing methods (e.g., application of filters) to detect and handle device non‐wear [5, 9, 10, 14, 19, 23, 30, 32, 43, 46, 49], and eight articles focused on detection and handling of data artifacts (e.g., non‐human movement) [20, 23, 38, 42, 43, 49, 50, 52, 53].

Ten articles discussed data reduction methods, recommending that authors describe the algorithms used to aggregate data [17, 27, 42, 46], as well as the (analysis) epoch length [5, 9, 20, 21, 23, 42, 46, 50]. One study additionally recommended reporting the rationale for this choice [42].

Six articles described reporting standards for studies where multiple types of data are collected (e.g., accelerometry and geolocation or heart rate), recommending that researchers describe how data sources are linked, steps taken to combine data, and how discrepancies between data sources are identified and handled [7, 14, 23, 38, 41, 47].

#### 3.3.3 Theme 3: Deriving Movement Behaviors From Acceleration Data

Theme three focuses on the methods used to derive behavioral variables or metrics after the initial processing of raw accelerometry data. This includes how acceleration values are classified into movement behaviors (e.g., sedentary time, physical activity, or sleep). Seven articles recommended that researchers should first identify the primary approach to analysis. This included clarifying whether the data are analyzed at the level of raw accelerometry signals, processed into summary measures (e.g., step counts, activity bouts) [34], or further modeled using predictive methods [17]. Authors should also describe the variable or metric of interest [5, 10, 23, 37, 47], and the units used to quantify this [47].

There was strong agreement across 24 articles that the details of algorithms, intensity cut points, scoring methods, or equations used to derive variables or classify behaviors should be clearly defined and reported [4, 5, 8, 9, 14, 15, 19, 20, 23, 27, 30, 32, 33, 38, 41, 42, 43, 45, 46, 47, 49, 50, 51, 54], that authors should justify their choice of methodology (e.g., whether individual or group/population specific or generic) [8, 17, 45, 46, 47] and acknowledge any limitations with the method used [8, 45, 46]. Clear definitions of behavioral variables were emphasized in nine articles [15, 19, 27, 34, 37, 41, 42, 47, 50], with examples including whether daytime sleep was included in total sleep time, describing how many days were included in the summary metric, and describing how bouts, breaks, or interruptions were accounted for. Reporting of software name, version, and settings was recommended in five articles, including default settings and when using commercial software [8, 15, 38, 46, 54].

Reporting of behavior classifications using advanced analytical methods such as machine learning, artificial intelligence, hot spot analysis techniques, and biomechanics was addressed in five articles [5, 23, 34, 38, 45]. Further details can be found in Additional File S4.

#### 3.3.4 Theme 4: Summary Metrics

The final theme concerns the summary outcomes, metrics, or variables authors recommend should be consistently reported to facilitate comparability across studies. Nine articles recommended specific reporting items relating to physical activity [22, 25, 34, 37, 42, 48, 51, 55], five articles suggested key metrics relating to sleep [7, 14, 18, 37, 52, 53], and three made recommendations for sedentary behavior metrics [19, 34, 35]. Full reporting items are listed in Additional File S4. Three articles also emphasized the importance of data transparency, recommending that raw datasets and processing/analysis code be made publicly available for future research [26, 30, 54].

There was some agreement across four articles that researchers should report average acceleration or count values (i.e., not influenced by cut‐points), to allow potential future reanalyzes using alternative methods [32, 39, 44, 48]. Three articles recommended that reporting should include both absolute (e.g., minutes per day) and relative (e.g., %) values for movement behaviors [34, 48, 55], with two also recommending reporting both central tendency and variability of the target behavior [19, 34]. Several articles recommended reporting temporal patterns of movement accumulation, such as the number and average duration of physical activity or sedentary bouts [19, 34, 37, 42]. Five articles suggested key metrics relating to sleep [7, 14, 18, 37, 52, 53], including total sleep time [7, 15, 18, 37, 52, 53], wake after sleep onset [7, 18], sleep efficiency [7, 14], and nap frequency and duration [7, 18].

## 4 Discussion

This scoping review provides an overview of recently published guidance relating to the information that should be reported when physical activity, sedentary behavior, and sleep have been assessed using accelerometers in the context of health research. The procedures for collecting and analyzing accelerometer data involve multiple steps, each with specific methodological choices. Several studies have demonstrated that these methodological decisions can significantly impact study outcomes and their interpretation, including estimation of the prevalence of individuals meeting physical activity guidelines [56, 57, 58, 59]. Ensuring consistency and transparency in reporting will enhance interpretability and reproducibility and also optimize the use of research resources [60].

Despite the variety of populations, device types, outcomes, and study designs considered in the 47 included articles, four key themes of recommendation emerged: data collection, data management and initial processing, deriving movement behaviors from acceleration data, and summary metrics. For data collection, most articles emphasized the importance of detailed reporting on the accelerometer model, device settings, and data collection protocols. For data management and processing, there was broad consensus on including wear‐time criteria, definitions of valid days, and how missing, incomplete, or implausible data, plus device removal, were identified and handled. It was also commonly recommended that studies specify the analytical epoch length and how data were filtered and processed before downstream analysis. For variable derivation, clear reporting of any algorithms, cut‐points, or scoring methods applied to the data, along with clear definitions of the variables derived, were widely recommended. Surprisingly, there was little guidance for reporting the results of performance evaluation (i.e., validation) of the classification algorithms used to derive variables of interest (e.g., steps, moderate‐vigorous physical activity, behavior types) supporting their selection, a major gap recently noted [61]. Transparent reporting in these areas ensures reproducibility and facilitates better cross‐study comparisons. As algorithms continue to evolve, particularly with the increasing use of advanced methods such as machine learning, clear justification of methodological choices and acknowledgments of their strengths and limitations are important to ensure the transparency and interpretation of findings. Several reporting guideline extensions are available for studies using artificial intelligence, including CONSORT‐AI (Consolidated Standards of Reporting Trials) [62], SPIRIT‐AI (Standard Protocol Items: Recommendations for Interventional Trials) [63] and TRIPOD‐AI (Transparent Reporting of a multivariable prediction model for Individual Prognosis Or Diagnosis) [64].

Whilst there was broad agreement on many reporting aspects, there was less guidance on which behavioral outcomes or metrics to report. The constant development of increasingly complex accelerometer‐based exposure and outcome metrics within health research makes it challenging to future‐proof recommendations for reporting. An extreme example of this includes applying deep learning techniques to directly predict health outcomes from raw accelerometer signals; this approach effectively identifies new features and motifs of the accelerometer trace that may not yet have a behavioral definition. In principle, comprehensive reporting of data collection, processing, and classification or prediction methods should ensure sufficient clarity for outcome reporting. This notion is supported by Crocker et al. [25], who recently sought to develop a core outcome set for RCTs evaluating physical activity interventions. However, given limited agreement on outcome domain inclusion and complexities of collecting, processing, and reporting device‐based data, a full core outcome set may not be applicable, and instead, guidance for standardized reporting of methods is needed.

In line with previous studies, we found that reporting guidance was developed using a variety of methodologies [65, 66]. Whilst most articles used an evidence‐based approach (e.g., systematic reviews), the level of stakeholder engagement in the development of reporting guidance was often unclear. Although some guidelines involved experts and opinion leaders, the nature of this involvement was poorly described. Robust guideline development depends on the consultation of diverse stakeholder groups to ensure relevance and applicability [12, 13, 66, 67]. These stakeholders include researchers, methodologists, healthcare professionals, journal editors, device manufacturers, software developers, and potentially patients or public representatives [13]. Their input can be valuable across multiple stages: (1) participating in modified Delphi studies and consensus meetings by rating, providing feedback, and ratifying proposed checklist items; (2) pilot testing and refining the guidance; and (3) promoting, endorsing, and applying the guidance in their respective domains. Following ACCORD recommendations will help ensure that these consensus processes are transparent and well‐documented [68].

The clarity and accessibility of reporting guidance also varied considerably across articles. Only some provided structured tables or figures to help communicate their recommendations. The EQUATOR approach for guidelines recommends providing explicit examples of good reporting practice, which would facilitate the accessibility of guidance for the health research community. Ultimately, reporting guidance must be practical and useful for different user groups, enabling academics to report, compare, and synthesize findings transparently; clinicians to interpret results for patient care; and patients and the public to understand the meaning and relevance of reported metrics.

### 4.1 Strengths and Limitations

To our knowledge, this scoping review is the most contemporary and comprehensive overview to date of reporting guidance for the application of accelerometry in health research. We adopted a broad scope, including reporting guidance for both interventional and observational research, and movement behavior outcomes that included sleep, sedentary behavior, and physical activity. To minimize bias, we applied a systematic review approach, including screening, data extraction, and thematic synthesis that involved agreement or checking by two reviewers. Although there is currently no recommended tool to assess the quality of reporting guidelines, we adapted both current guidance for developing reporting guidelines [13], and the AGREE II tool for assessing the quality of practice guidelines [12], to examine the methods employed to develop the identified reporting guidance. However, we recognize our study has limitations. First, we did not search gray literature. Second, many of the recommendations were extracted from publications that did not explicitly identify as guidance development studies. Third, we may have missed or potentially misinterpreted some items in the included guidance documents, highlighting that ambiguity in terminology may in itself also be a limitation.

### 4.2 Future Research

A significant volume of reporting recommendations was identified across the included articles in this scoping review. This emphasizes the continuing need for consolidated, standardized reporting guidance for accelerometer‐based research. Consensus on such guidance should be achieved using a transparent and structured process, as recommended by the EQUATOR network [13]. Cross‐disciplinary and cross‐institutional collaboration will be important to ensure relevance and broad applicability. Ongoing initiatives such as ProPASS and ISPAH demonstrate how coordinated efforts can successfully align methodological standards and promote harmonization [69, 70].

The practical application of accelerometry reporting guidance will vary depending on study design and purpose. Certain items, such as device specifications, wear protocols, and data processing methods are likely to be relevant across observational, interventional, and surveillance studies. Other items may be more context‐specific. For example, while this review excluded guidance focused solely on device validation or calibration studies, we acknowledge that some recommendations (e.g., device placement protocol) may still be applicable to those contexts. Future guidance should aim to distinguish between core reporting items that are universally relevant and context‐specific items tailored to particular study designs or objectives. This approach will support the development of flexible and adaptable frameworks that can be applied across diverse research settings.

To encourage uptake, future reporting guidance should be embedded within journal author and peer reviewer instructions, an approach that has been proven effective for other reporting standards. Widely endorsed frameworks such as CONSORT [71], Strengthening the Reporting of Observational studies in Epidemiology (STROBE) [72], and SPIRIT [73] have significantly improved the quality of reporting in clinical and epidemiological research [74]. Integrating accelerometry‐specific extension into these established guidelines could similarly enhance transparency and consistency of reporting in health research.

Building on the reporting guidance items and gaps identified in this scoping review, we have recently begun an online modified Delphi process to agree on items for the development of future guidance in this field. Our aim is to produce CONSORT, STROBE, and SPIRIT extensions tailored to accelerometry use in RCTs and observational studies [75].

### 4.3 Perspective

This scoping review identified existing reporting guidance for using accelerometry to assess physical activity, sedentary behavior, and sleep. Our review indicates that there is some consistency in reporting guidance related to data collection, data processing, and variable derivation methods. However, outcome reporting poses more of a challenge. Additionally, the methodology employed by authors to develop previous reporting guidance was highly variable, with limited transparency around stakeholder involvement. Clear and consistent reporting is essential to ensure transparency, reproducibility, and comparability across studies using accelerometry. Without it, the potential of accelerometry to advance our understanding of movement behaviors and their health impacts may be limited. A more systematic and rigorous approach to future guideline development, inclusive of diverse stakeholder perspectives, is needed to support the continued growth and impact of accelerometry in health research.

## Author Contributions

G.O.D., S.B., M.B., E.D., C.F., M.H.G., M.H., A.M., C.E.M., P.M., R.S.T., and T.V. developed the concept and protocol for the study. G.O.D. and C.S. led the study selection and extraction. G.O.D. and C.F. led the data synthesis and analysis. G.O.D. prepared the initial draft. All authors reviewed the manuscript.

## Ethics Statement

The authors have nothing to report.

## Consent

The authors have nothing to report.

## Conflicts of Interest

The authors declare no conflicts of interest.

## Supporting information


**Appendix S1:** sms70143‐sup‐0001‐AppendixS1.pdf.

## Data Availability

Data sharing is not applicable to this article as no new data were created or analyzed in this study.
