# Supplementary material for: Reporting of Accelerometry in Health Research: A Scoping Review of Current Guidance
Source: Scand J Med Sci Sports. 2025 Oct 15;35(10):e70143. doi: 10.1111/sms.70143 (PMC12525895; doi:10.1111/sms.70143)
Supplement: Supplementary file 1 — Appendix S1: sms70143‐sup‐0001‐AppendixS1.pdf. [file SMS-35-e70143-s001.pdf]

Additional files

Contents

Additional file 1: PRISMA-ScR checklist 2
Additional file 2: Search strategy 4
Additional file 3: Summary of stakeholder involvement and consensus activities 5
Additional file 4: Summary of reporting recommendations 8

Additional file 1: PRISMA-ScR checklist

Preferred Reporting Items for Systematic reviews and Meta-Analyses extension for Scoping Reviews (PRISMA-ScR) Checklist

SECTION	ITEM	PRISMA-ScR CHECKLIST ITEM	REPORTED ON PAGE #
**TITLE**			
Title	1	Identify the report as a scoping review.	1
**ABSTRACT**			
Structured summary	2	Provide a structured summary that includes (as applicable): background, objectives, eligibility criteria, sources of evidence, charting methods, results, and conclusions that relate to the review questions and objectives.	2-3
**INTRODUCTION**			
Rationale	3	Describe the rationale for the review in the context of what is already known. Explain why the review questions/objectives lend themselves to a scoping review approach.	4-5
Objectives	4	Provide an explicit statement of the questions and objectives being addressed with reference to their key elements (e.g., population or participants, concepts, and context) or other relevant key elements used to conceptualize the review questions and/or objectives.	4-5
**METHODS**			
Protocol and registration	5	Indicate whether a review protocol exists; state if and where it can be accessed (e.g., a Web address); and if available, provide registration information, including the registration number.	5
Eligibility criteria	6	Specify characteristics of the sources of evidence used as eligibility criteria (e.g., years considered, language, and publication status), and provide a rationale.	6-7, Table 1
Information sources*	7	Describe all information sources in the search (e.g., databases with dates of coverage and contact with authors to identify additional sources), as well as the date the most recent search was executed.	5-6
Search	8	Present the full electronic search strategy for at least 1 database, including any limits used, such that it could be repeated.	Additional file 2
Selection of sources of evidence†	9	State the process for selecting sources of evidence (i.e., screening and eligibility) included in the scoping review.	6
Data charting process‡	10	Describe the methods of charting data from the included sources of evidence (e.g., calibrated forms or forms that have been tested by the team before their use, and whether data charting was done independently or in duplicate) and any processes for obtaining and confirming data from investigators.	7-8
Data items	11	List and define all variables for which data were sought and any assumptions and simplifications made.	7-8
Critical appraisal of individual sources of evidence§	12	If done, provide a rationale for conducting a critical appraisal of included sources of evidence; describe the methods used and how this information was used in any data synthesis (if appropriate).	7-8

SECTION	ITEM	PRISMA-ScR CHECKLIST ITEM	REPORTED ON PAGE #
Synthesis of results	13	Describe the methods of handling and summarizing the data that were charted.	8
**RESULTS**			
Selection of sources of evidence	14	Give numbers of sources of evidence screened, assessed for eligibility, and included in the review, with reasons for exclusions at each stage, ideally using a flow diagram.	8, Figure 1
Characteristics of sources of evidence	15	For each source of evidence, present characteristics for which data were charted and provide the citations.	Table 2, Reference list
Critical appraisal within sources of evidence	16	If done, present data on critical appraisal of included sources of evidence (see item 12).	9-11
Results of individual sources of evidence	17	For each included source of evidence, present the relevant data that were charted that relate to the review questions and objectives.	Additional file 4
Synthesis of results	18	Summarize and/or present the charting results as they relate to the review questions and objectives.	11-15
**DISCUSSION**			
Summary of evidence	19	Summarize the main results (including an overview of concepts, themes, and types of evidence available), link to the review questions and objectives, and consider the relevance to key groups.	15-18
Limitations	20	Discuss the limitations of the scoping review process.	18
Conclusions	21	Provide a general interpretation of the results with respect to the review questions and objectives, as well as potential implications and/or next steps.	19
**FUNDING**			
Funding	22	Describe sources of funding for the included sources of evidence, as well as sources of funding for the scoping review. Describe the role of the funders of the scoping review.	28

JBI = Joanna Briggs Institute; PRISMA-ScR = Preferred Reporting Items for Systematic reviews and Meta-Analyses extension for Scoping Reviews.

* Where *sources of evidence* (see second footnote) are compiled from, such as bibliographic databases, social media platforms, and Web sites.

† A more inclusive/heterogeneous term used to account for the different types of evidence or data sources (e.g., quantitative and/or qualitative research, expert opinion, and policy documents) that may be eligible in a scoping review as opposed to only studies. This is not to be confused with *information sources* (see first footnote).

‡ The frameworks by Arksey and O'Malley (6) and Levac and colleagues (7) and the JBI guidance (4, 5) refer to the process of data extraction in a scoping review as data charting.

§ The process of systematically examining research evidence to assess its validity, results, and relevance before using it to inform a decision. This term is used for items 12 and 19 instead of "risk of bias" (which is more applicable to systematic reviews of interventions) to include and acknowledge the various sources of evidence that may be used in a scoping review (e.g., quantitative and/or qualitative research, expert opinion, and policy document).

From: Tricco AC, Lillie E, Zarin W, O'Brien KK, Colquhoun H, Levac D, et al. PRISMA Extension for Scoping Reviews (PRISMAScR): Checklist and Explanation. Ann Intern Med. 2018;169:467–473. doi: 10.7326/M18-0850.

Additional file 2: Search strategy

PsycINFO search

#	Query
S6	S3 AND S4 Limit to 2014-2021
S5	S3 AND S4
S4	S1 AND S2
S3	TI (Guideline* or Reporting or Publishing or Publication or Consensus) OR AB (Guideline* OR Reporting or Publishing or Publication or Consensus)
S2	TI ((Guide* or Checklist* or Recommendation* or Standard* or Requirement* or Instruction* or Policy or Quality or Template or Method* or Advice or "Good practice" or "Best practice" or "Standard practice" or Criteria or Approach or Framework or Tool or Validation or validity or Reliability OR transparency)) OR AB ((Guide* or Checklist* or Recommendation* or Standard* or Requirement* or Instruction* or Policy or Quality or Template or Method* or Advice or "Good practice" or "Best practice" or "Standard practice" or Criteria or Approach or Framework or Tool or Validation or validity or Reliability OR transparency))
S1	TI (Acceleromet* or Actig*)) OR AB (Acceleromet* or Actig*))

Additional file 3: Summary of stakeholder involvement and consensus activities

Study	Stakeholder description – who? How many?	How were stakeholders involved?	Consensus activity description and results
Ancoli-Israel (2015)	**Who**: "Experts in the field of actigraphy and sleep"<br>**How many**: Not specifically described, but 9 authors listed.	Not described – "The content was developed by"… "and represents the current "state of the science" in the use of wrist actigraphy for clinical populations."	Not done
Crocker (2022)	**Who**: "academics with an interest in physical activity or outcomes measurement; professionals working in the field of public health; health care professionals; and lay people aged 18 or over." Professionals were at various career stages, but skewed towards those holding higher positions.<br>**How many**:<br>Delphi 1:<br>41 (36 professionals, 5 lay people) invited to take part. 20 (16 professionals, 4 lay people) completed round 1.<br>36 (32 professionals, 4 lay people) were invited to take part. 21 (17 professionals, 4 lay people) completed round 2 (17 of whom participated in round 1).<br>Delphi 2:<br>36 (same participants as round 2 above) invited to participate. 19 (15 professionals, 4 lay people) completed the survey.	Invited to participate in a series of electronic Delphi surveys.<br><br>Delphi survey 1:<br>Purpose: Reach agreement on the outcome domains for the core outcome set.<br>Process: presented a list of 13 outcome domains commonly measured in clinical trials of physical activity. Asked to rate the importance of each domain on a scale of 1-9, where 1-3 signified limited importance, 4-6 was important but not critical, and 7-9 was critical importance. In the second round, participants presented with a summary of results from round 1 (the average rating for each domain and the distribution of ratings) and asked to re-rate the outcome domains.<br><br>Delphi survey 2: participants given a report detailing the study background, previous results and brief summaries of the literature prior to completing the second survey. The second survey consisted of 6 questions, rating a list of device-based measures of physical activity from 1 (do not recommend) to 5 (highly recommend), and remaining questions asking participants whether they agreed with a series of statements about the included outcome domains and a further proposed outcome domain.	Two Delphi surveys. Consensus to include considered to be met if ≥70% of participants rated a domain between 7-9 and <15% rated between 1-3. Consensus to exclude considered to be met if ≥70% of participants rated a domain between 1-3 and <15% rated between 7-9.<br><br>Round 1: consensus was reached for 2 outcome domains to be included in the core outcome set (level of physical activity, and health related quality of life). No consensus was reached for the remaining 11 outcome domains. No new outcome domains were added for consideration in round 2.<br><br>Round 2: no consensus was reached for the remaining 11 outcome domains.<br><br>Survey 2:<br>Respondents rated the accelerometer as the best device to include in the core outcome set for measuring level of physical activity.
Demeyer (2021)	**Who**: "The task force included a global panel of key opinion leaders from the field as well as key industry partners conducting research in COPD	Pooled data from existing studies to answer key methodologic questions. Included studies from individual	Not done

	with physical activity endpoints."<br>**How many**: Not specifically described, but 32 authors listed.	investigator members of the consortium.	
Depner (2020)	**Who**: "experts in consumer sleep technologies, medical devices, sleep and circadian physiology, clinical translational research, and clinical practice." "Participants of the workshop included members of the Sleep Research Society, American Academy of Sleep Medicine, Asian Society of Sleep Medicine, Australasian Sleep Association, Canadian Sleep and Circadian Network, Canadian Sleep Society, Canadian Society for Chronobiology, Cerebra, Department of Defense of the United States of America, European Sleep Research Society, f.Lux, National Aeronautics and Space Administration of the United States of America, National Institutes of Health of the United States of America, National Center for Sleep Disorders Research of the United States of America, Rythm, and Society for Research on Biological Rhythms."<br>**How many:** not described – 8 authors and "on behalf of…"	Attended a workshop, "International Biomarkers Workshop on Wearables in Sleep and Circadian Science" in 2018.<br><br>Workshop participants discussed barriers and opportunities for using wearables in sleep and circadian science. Poor validation of current wearable technology was identified as the primary barrier inhibiting widespread use of wearables in sleep and circadian science. The committee proposed a set of best practices for validation studies to help overcome this barrier.	Workshop consensus opinions reported throughout paper, but process to reach consensus not described.
Ellender (2024)	**Who:** "a subcommittee of experienced sleep physicians and scientists" empaneled by the Australian Sleep Association.<br>**How many:** not described – 10 authors	Subcommittee were tasked to review the literature and formulate recommendations on the indications, performance, and reporting of sleep studies, to update clinical practice from the 2017 Australasian Sleep Association (ASA) guidelines for sleep studies in adults.	Document described as a consensus statement – process to reach consensus not described.
Gabrys (2015)	**Who**: Renowned scientists in German-speaking countries, German-speaking working groups and individual authors with proven publications in the subject area<br>**How many**: not described – 13 authors	Recommendations include results of the expert workshop held as part of the 21st German University Congress of the German Association for Sports Science and the expertise of recognised scientists.	The recommendations were developed by circulation and adopted by consensus. No further details provided.

		The recommendations were made after screening literature, developed by circulation, specified and adopted by consensus.	
Lee (2021)	**Who**: Authorship team **-** "Multidisciplinary team (physiotherapists, occupational therapists, software engineer and biomechanist) to ensure that a diverse range of knowledge and expertise was utilised."<br>**How many:** 6 authors	Conducted the scoping review	Not done.
Migueles (2021)	**Who**: A panel of European researchers selected from the speaker and participant list of the GRANADA workshop: 'a focus on statistical methods to analyse accelerometer-measured physical activity'. Manuscript reviewed by external experts in the field from different countries, including Europe, USA and Australia<br>**How many**: workshop attendees not described – 14 authors, 11 collaborators listed as the "External review group"	Attended the 'International Workshop: A focus on statistical methods to analyse accelerometer-measured PA' held in Granada, October 2019. Researchers discussed, reached consensus and provided recommendations<br><br>Additionally, the manuscript was sent for review to external experts in the field.	Described as a consensus manuscript – process to reach consensus during workshop not described.<br><br>More than 150 comments received from panel of external reviewers.
Van Hees (2016)	**Who:** Attendees at the Graduate School for Information Science in Health (GSISH) Biosensor workshop in 2014, with invitees from the field of physical activity assessment. "The workshop attendees are the authors of this article", representing expertise in sensor manufacturing, signal processing, study design, doubly labelled water studies, clinical studies and population-based research.<br>**How many:** 12 authors	"The workshop included plenary discussions as well as subgroup discussions."<br><br>Participants discussed generic aspects of the raw accelerometry harmonisation process (who are the stakeholders, how are methodological standards established, how does harmonisation relate to the fundamental principles of scientific transparency and openness?)<br>Participants then discussed aspects of raw accelerometry methodology: hardware, data collection and study protocols, method development and evaluation, data processing, data description before and after processing, method implementation and evaluation.	Not done

Additional file 4: Summary of reporting recommendations

Main theme	Subtheme	Summary of recommendations (references)	No. of articles
1. Data collection	1.1 Device(s) specifications	Details of the device including:<br>• Name (17, 19, 27, 46, 47, 50, 51)<br>• Model (5, 9, 10, 14, 17, 23, 37, 38, 49, 50)<br>• Device version number (27, 50)<br>• Manufacturer (5, 10, 14, 19, 23, 27, 37, 38, 49, 50)<br>• Firmware version (24, 29)<br>• Type (e.g. accelerometer/gyroscope, heart rate, other) (17, 38, 54)<br>• Number of axes (54)<br>• Unique accelerometer ID numbers (52/53)<br>• Battery time (26)<br>• Weight (26)<br>• Size (26)<br>• Price (26)	**20**
		If multiple devices or concurrent logs were used<br>• Provide details (14, 42, 50)<br>• Rationale for the number of devices (42)	3
		• Indication or rationale for using the device (7, 42)	2
		• Validity and reliability of the device (17, 27, 38, 41, 42, 47, 49, 51)	8
		If mobile phone (17):<br>• Whether the device was provided to the participant or they used their own<br>• study app name<br>• which sensors were used, e.g. accelerometer, GPS, ambient light, microphone<br>• phone analytics collected, e.g. apps used, screen unlock/lock, Bluetooth connections, cell tower connections.<br>• Types of passive data used (e.g., phone sensors, phone use analytics)	1
		If the device provides the wearer feedback, details should be provided (38):<br>• Trigger (kinematic set-point)<br>• Biomechanical set point source/origin<br>• Content (visual, audible, vibrotactile, multimodal, other)<br>• Timing (latency) (concurrent, terminal, fading, other)<br>• Frequency of feedback occurrences	1

		• Monitoring duration<br>• Source/device of feedback<br>• Participant evaluation on feedback content/timing	
	1.2 Settings and configurations at device initialisation (including default settings within the device software)	• Epoch length – during data collection – any onboard data reduction? (5, 10, 14, 20, 21, 32, 43, 46, 47, 49, 50, 51)	12
		• Sampling rate/frequency (9, 14, 20, 23, 26, 27, 32, 37, 38, 43, 47, 49, 54)	13
		• Whether idle sleep mode was enabled (23)	1
		• Data filters or filter settings (5, 20, 43, 46, 51)	5
		• Sensor ranges (5)	1
	1.3 Data collection protocol	Placement of device on the participant (5, 9, 10, 20, 23, 32, 37, 38, 42, 43, 46, 49, 51, 54), specifically:<br>• site and side of body, (5, 10, 38, 49)<br>• orientation of device, (5)<br>• attachment method (5, 38, 49)	14
		Data collection period:<br>• Detail the data collection period or wear time goal, e.g. number of days, whether waking hours or 24-hour period, weekdays or weekend days (5, 7, 10, 14, 17, 19, 20, 27, 32, 36, 42, 46, 47, 49, 55)<br>• Rationale for the length of time being sufficient to give a good estimate of target behaviours. (34, 42, 55)	16
		• Relevant setting or context details such as time of year of measurement, weather, or the location of the participant (e.g. at work, home, hospital or institution). (14, 17, 23, 47, 51)	5
		• Details of the device distribution and collection methods e.g. number of accelerometers distributed, retrieved and how [i.e. face-to-face or postal] (5, 10, 38, 42, 46, 49)	6
		• Instructions given to participants – such as information on proper wear, removal whilst bathing, water-based activities or sleeping (27, 31, 37, 42, 49)<br>• whether instructions were translated (37)	5
		• Behavioural incentives or strategies employed to monitor and encourage compliance – e.g. logs or diaries, reminder calls, incentives. (17, 37, 42, 49, 51)	5
		Participant recruitment and device distribution:<br>• Number of participants invited to wear an accelerometer (49, 52/53)	4

		• Number of withdrawals (declined to wear accelerometer after consent) (49, 52/53)<br>• Number of devices distributed (5, 10, 49, 52/53)<br>• Accelerometer capacity (i.e. the mean number of participants assessed per accelerometer) (52/53)<br>• Number of devices lost during data collection, (49, 52/53)	
	1.4 Other details	• Whether the sample size is sufficient to give a good estimate of the target behaviour of the population (34)	1
		• Data privacy laws and regulations around study (17)	1
		• Levels of participants' digital technology literacy (17)	1
2. Data management and initial processing	2.1 Valid data sets	• Criteria for determining whether data is valid for analysis (e.g., minimum wear time, required number of valid days) (5, 10, 19, 20, 21, 22, 27, 30, 32, 34, 36, 37, 41, 42, 46, 49, 55)	17
		• Number of valid data sets obtained, or participants included in the final analysis (5, 14, 17, 21, 23, 36, 46, 49, 47, 51, 52/53)	11
		• Summary of average or total wear time, or adherence (17, 19, 21, 22, 26, 32, 36, 37, 46, 49, 51, 52/53, 55)	13
	2.2 Data completeness	Missing data:<br>• Procedures for identifying and handling missing data (e.g. imputation) (16, 30, 37, 42, 47, 49, 50)<br>• Overall rate of missing data (16, 17, 23, 35, 47)<br>• Assessment of the randomness of missingness (35) or response bias, i.e., differences between invited participants and those in the final analytical sample (49)<br>• Number of participants with imputed data included in the final analytical sample (49)<br>• Whether participants with missing data were given an opportunity to repeat the assessment and how many did (49)	10
		Reasons for data loss or exclusions (40, 52/53):<br>• Number of data sets excluded due to invalid data (5, 10, 14, 37, 49, 51, 52/53)<br>• Data loss due to technical problems with device initialisation, data extraction or other malfunctions (5, 7, 14, 49)	9
		• Limitations associated with interpreting results relating to missing data, or technical factors of concern (7, 14, 35)	3

	2.3 Data cleaning and filtering	• Method used to detect and handle non-wear time or device removal (e.g., manually or automatic detection, handling in analysis) (5, 9, 10, 14, 19, 23, 30, 32, 43, 46, 49)	11
		• Detection and handling of data artifacts (e.g., non-human movement signals; filter applied, data excluded) (20, 23, 38, 42, 43, 49, 50, 52/53)	8
	2.4 Data reduction/ aggregation	• Algorithms used for data aggregation (e.g., daily summary, bout analysis, vector) (17, 27, 42, 46)<br>• Epoch length (5, 9, 20, 21, 23, 42, 46, 50) and rationale (42)	10
	2.5 Data linkage	Where multiple types of data are collected (e.g., accelerometer and GPS)<br>• Description of how multiple data sources were linked (e.g., temporal or spatial) (41, 47)<br>• Steps taken to combine data from different sources (23, 38, 47)<br>• Discrepancies between concurrent devices/logs, and how identified and handled (7, 14)	6
3. Deriving movement behaviours from acceleration data	3.1 Main analysis approach	Identify the main approach to analysis:<br>• Type of analysis (e.g., inferential statistics or predictive machine learning) (17)<br>• Data processing approach: Are data analysed using accelerometer counts or pattern recognition algorithms? (34)<br>• Behavioural variable or metric of interest? (5, 10, 23, 37, 47)<br>• Units used to quantify the metric (47)	7
	3.2 Computing variables	Methods for computing variables:<br>• Cut-points / algorithms / classification methods / scoring methods / equations applied (4, 5, 8, 9, 14, 17, 19, 20, 23, 27, 30, 32, 33, 38, 41, 42, 43, 46, 47, 49, 50, 51, 54) including source and developer (8)<br>• Justification for choice of method (8, 17, 45, 46, 47) with reference to other publications that have used the same methods, and support the appropriateness of its application, where possible (8)<br>• Acknowledgement of limitations (45, 46)<br>• Clear description or definition of derived metrics, e.g. whether daytime sleep is included total sleep time calculations? Does sedentary time include sleep? How many days are included in the summarised outcome (weekend, or weekdays?) (27, 34, 41, 47, 50)<br>• Definitions of bouts, breaks or interruptions and how they were accounted for (15, 19, 42, 37)<br>• Performance reporting – efficiency, accuracy of the algorithm or analysis (17, 30)	26

	3.3 Software	Details of the software used to derive the variable, including:<br>• Name (8, 14, 38, 46, 54)<br>• Version (14)<br>• Settings (default settings if using commercial software) (8, 14)	5
	3.4 Advanced analytical methods	Some specific details were identified for advanced approaches including<br>• Machine learning: Features extracted, window length, parameter choices, specific model information (5,8)<br>• Use of pattern recognition algorithms or inclinometry (34)<br>• Signposting to TRIPOD or TRIPOD-AI checklists (45)<br>• Hot spot analysis: Distance band, time band, weighting scheme (23)<br>• 3D joint/modelling angle(s)/rotation(s) – refer to International Society of Biomechanics, or provide equivalent information to replicate (38)	6
4. Summary metrics	General	• Report absolute (hours and mins/day) and relative (%) values (34, 48, 55)<br>• Report both central tendency and variability of target behaviour (19, 34)<br>• Report metrics or raw values that are not influenced by cut-points to allow future reanalyses using alternative methods (32, 39, 44, 48)<br>• Carefully consider the use of guidelines as an outcome measure, and acknowledge the rationale and limitations of doing so (46)<br>• Present findings disaggregated by key research questions, such as gender or work/non-work time (34)<br>• Adverse events relating to accelerometer device (26, 49)<br>• Acceptability of device (26, 28)	11
	Physical activity	• Several measures of PA should be reported, with examples including step count, sedentary activity, acceleration levels, energy expenditure, and activity intensity (22, 51)<br>• Total duration or volume of PA per day (25, 34, 37, 42, 55)<br>• Time spent in intensity ranges (32, 51)<br>• Percentage of day spent in PA (32, 37)<br>• Number of PA bouts per day (42)<br>• Number of continuous PA sessions by level of effort (37)<br>• Average duration of PA bouts (34, 42)<br>• Total step count (37, 51)<br>• Raw counts per minute (48, 51)	9

		• METS, if applicable (51)	
	Sleep	• Sleep onset (14)<br>• Sleep offset (14)<br>• Sleep period (14)<br>• Total sleep episode time (7, 14, 18, 37, 52/53)<br>• Total wake time (14)<br>• Average waking time (37)<br>• Sleep efficiency or sleep percent (7, 14)<br>• Sleep latency (7, 18)<br>• Wake after sleep onset (7, 18)<br>• Awakening (frequency) (18)<br>• Nap duration (7, 18)<br>• Nap frequency (7, 18)<br>• Presence of circadian rhythm shift (7)<br>• Inter-day variability/stability (7)	5
	Sedentary behaviour	• Total daily duration of sedentary time (19, 34, 37)<br>• Number of bouts of sedentary behaviour (19)<br>• A metric describing the diversity of bout length in the sedentary behaviour (19)	3
	Data transparency	• Make raw datasets available for future research (26, 54)<br>• Make code publicly available or usable (30, 54)	3
